# Nature As a “Lifeline”: The Power of Photography When Exploring the Experiences of Older Adults Living With Memory Loss and Memory Concerns

**DOI:** 10.1093/geront/gnad126

**Published:** 2023-10-04

**Authors:** Elenyd Whitfield, Sukey Parnell Johnson, Paul Higgs, Wendy Martin, Sarah Morgan-Trimmer, Alexandra Burton, Michaela Poppe, Claudia Cooper

**Affiliations:** Centre for Psychiatry and Mental Health, Wolfson Institute of Population Health, Queen Mary University of London, London, UK; Faculty of Medical Humanities, Kings College London, London, UK; UCL Division of Psychiatry, University College London, London, UK; Department of Health Sciences, Brunel University London, LondonUK; Department of Health and Community Science, University of Exeter, Exeter, UK; Department of Behavioural Science and Health, University College London, London, UK; Centre for Psychiatry and Mental Health, Wolfson Institute of Population Health, Queen Mary University of London, London, UK; Centre for Psychiatry and Mental Health, Wolfson Institute of Population Health, Queen Mary University of London, London, UK

**Keywords:** Collaborative, Mild cognitive impairment, Participatory photography, Subjective cognitive decline

## Abstract

The visual is an underutilized modality through which to investigate experiences of memory loss in older people. We describe a visual ethnography with older adults experiencing subjective or objective memory loss, receiving a cognitive well-being group intervention designed to prevent cognitive decline and dementia (APPLE-Tree program). We aimed to explore lived experiences of people with memory concerns, how participants engaged with this photography and codesign project, and how collaboration with an artist/photographer enhanced this process. Nineteen participants shared photographs reflecting what they valued in their daily lives, their experiences of memory concerns, and the intervention. Fourteen participated in qualitative photo-elicitation interviews, and 13 collaborated with a professional artist/photographer to cocreate an exhibition, in individual meetings and workshops, during which a researcher took ethnographic field notes. Eight participants were reinterviewed after the exhibition launch.We contextualize images produced by participants in relation to discourses around the visual and aging and highlight their relationship with themes developed through thematic analysis that interconnects photographic, observational, and interview data. We present themes around the use of photographs to: (1) celebrate connections to nature as a lifeline; (2) anchor lives within the context of relationships with family; and (3) reflect on self and identity, enduring through aging, memory concerns, pandemic, and aging stereotypes. We explore visual research as a powerful tool for eliciting meaningful accounts from older adults experiencing cognitive change and to connect the arts and social sciences within aging studies.

Cultural gerontology is characterized by explorations of social identity, subjective experiences, and cultural depictions of aging, agism, and embodiment (Andersson, 2002; Twigg & Martin, 2015a, 2015b), for which visual methods are powerful and underused tools. Older bodies are often seen as “a disruption to the visual field” in youth-oriented societies (Hepworth, 2000). Visual research methods resist this (Hogan & Warren, 2012; Martin, 2015); images are cultural objects and are “at least potentially a site of resistance and recalcitrance, of the irreducibly particular” (Armstrong, 1996, p. 28 see also Rose, 2001, p. 11). Images of aging are not merely illustrative but have a “visual autonomy and specific mediality” (Kampmann, 2015, p. 279).

Cultural gerontology highlighted not only the arrival of the humanities into aging studies but also increased interconnections between the social sciences and arts; artists and social scientists collaboratively bringing novel perspectives and methodologies (Twigg & Martin, 2015a, 2015b). Discussions around the conjunction between photography and aging from an arts and humanities perspective have often focused on images of older people and the physicality of aging. These images and discussions are important to counteract the relative invisibility of older people in popular culture (Cristofovici, 2009), to explore the “double-standard” of gendered attitudes to physical aging (Pilcher & Martin, 2020; Sontag, 1979; Woodward, 1999), and to examine polarizing tendencies in representation in images of older people; “decline” versus “positive aging” (Featherstone & Wernick, 1995) or “melancholic” versus “carnivalesque” (Parnell Johnson, 2018; Richards et al., 2012, drawing on Parnell Johnson).

Cristofovici asks how photographers depict the “inner realities” of aging (2009, pp. 20–21). The relationship between aging and photography is not confined only to images of older people (Cristofovici, 2021). In this study, the images were taken mostly by older people, and mostly not of them, depicting their inner realities through their selection of photographs to best represent their lives. This contrasts with projects where older people are mostly subjects of the image, taken by photographers who may be considerably younger than them (Richards et al., 2012).

The research method of participatory photography responds to criticisms of “inequity of power, the politics of representation and the objectification of the other” (Clover, 2006, pp. 275–276). It empowers participants to represent their realities (Clover, 2006; Reyes et al., 2022, p. 4) and explore self and subjectivity (Pink et al., 2011). Images, and participants’ responses to them, are not simply more data but different data; tools to elicit insights and meanings (Harper, 2002, p. 99), which allow “different and more textured understandings of ageing identities” (Martin, 2015, p. 100).

Participant-generated images have been used in previous studies with older people, including people with mild cognitive impairment (MCI; Renn et al., 2021). Brotherhood et al.’s *Created Out of Mind* residency codesigned multiple performances and exhibitions with participants with dementia (2017). Although other participatory photography projects with older people have incorporated workshops and exhibitions (e.g., Hogan & Warren, 2012; Martin, 2015; Reyes et al., 2022), our project is, we believe, the first to report on a sustained engagement in which an artist/photographer, social science researchers, and participants with memory concerns, worked together in different modalities (interview, zoom sessions, workshop, exhibition cocreation). The timing of this work in relation to the coronavirus disease 2019 (COVID-19) pandemic provides an interesting context.

We aimed to explore how participants engaged with a photography and codesign project to convey their lived experiences of memory loss and dementia prevention, and how this creative and reflective process was enhanced by collaboration between social scientists, participants with lived experience, and an artist/photographer who specializes in the representation of aging. Our analysis of the data generated through this was shaped by the engagement of participants and the types of images they shared with us.

## Method

### Recruitment

We recruited participants aged 60 and older with subjective cognitive decline or MCI from the APPLE-Tree Randomized Controlled Trial, which evaluated a dementia prevention program involving weekly small groups, interactive video-call sessions, and phone calls with facilitators (Cooper et al., 2021; Poppe et al., 2022). We recruited from across the intervention arm groups, promoting the study through emailed “leaflets” and visits to video-call sessions, with attention paid to cultural and ethnic diversity. Participants were invited to take part in either, or both, photo-elicitation interviews and collaboration with a professional artist/photographer to codesign an exhibition. No incentives were offered for participation.

### Data Collection Methods

E. Whitfield (social science researcher) conducted photo-elicitation interviews between March and September 2022. S. Parnell Johnson (artist/photographer) and E. Whitfield facilitated exhibition codesign, in individual and group sessions, between April and August 2022. After the exhibition, E. Whitfield conducted further interviews.

#### Photo-elicitation interviews

Following established methods of using participant-generated photographs in photo-elicitation interviews (Balomenou & Garrod, 2016), E. Whitfield invited participants to take a series of photographs (around 15 for more than 1–2 weeks) that felt relevant to their memory concerns and participation in the well-being program. Photographs could include objects, activities, images of people, views, or more abstract images. We intended this guidance to be orientating but not overly directive, to empower participants to generate their own meanings and representational style of photograph. Some participants shared more or less than 15 photos (the highest number was 50), and some included photos that had been taken previously, including some images of them taken by others. Images were mostly taken with camera phones or occasionally an iPad, and some with participants’ own cameras.

E. Whitfield conducted semistructured interviews with participants, mostly in their homes, two by video-call. She invited participants to share and discuss photographs taken, and a topic guide shaped discussions of their experiences of memory concerns, broader lives, and identities. This included asking participants about their involvement in APPLE-Tree and if they had spoken to friends and family about having concerns about their memory. Interviews were audio-recorded and transcribed.

The use of interview prompts following discussion of photos follows methods used by Klingorová and Gökarıksel (2019).

#### Collaborating with an artist/photographer and cocreating an exhibition

Participants in this part of the project met with S. Parnell Johnson and E. Whitfield individually through video-call sessions once or twice before they attended small-group workshops, depending on what felt useful and possible for them. Timings varied but were usually for around 30–60 min. Participants provided photographs before sessions, including those explored in photo-elicitation interviews, and additional photographs that spoke to “things that are important to me.”

S. Parnell Johnson led these video-call sessions discussing images, experiences, and stories participants wished to convey to audiences, and exhibition work (photographic arrangements) they wanted to develop; E. Whitfield took observational fieldnotes. S. Parnell Johnson used appropriate software to display and explore images and advised on lighting and other technical considerations. This online visual method was developed by her in her work with students during the COVID lockdown. The kinetic visual element was critical, enabling her to move images around in dialogue with participants, making new groupings/arrangements/sequences that prompted different connections. Most participants used their phones or own cameras and two borrowed cameras from the project.

Next, we held four half-day workshops (three in person, one video-call group) for four or five participants each. Developed photographs and printed arrangements from initial video-calls were given to participants at the beginning of the face-to-face workshops. Photographs were sent to virtual workshop participants after the session. Participants responded to each other’s photos, wrote sticky notes on their own to describe meanings, arranged photographs, and wrote accompanying text. E. Whitfield and S. Parnell Johnson continued to support participants to develop their work after the workshops through email and video-call, and in some instances for E. Whitfield to further explore their thoughts, feelings, and reflections on the images. Participants had a designated space on the exhibition display boards and were invited to display photos and text. S. Parnell Johnson collaborated with participants on how to present work creatively, suggested other types of display for some sets of photos, and laid out the designs using appropriate software, to be printed onto large boards.

Next, we organized two coproduction meetings to plan the exhibition and accompanying film and catalog, with four and five participants each, and study team members (academics and a Public and Patient Involvement representative). S. Parnell Johnson presented images and initial overarching exhibition themes that she and E. Whitfield had developed, and the group discussed the meanings behind their images. The second meeting included a visit to the exhibition site.

### Ethics

London (Camden and Kings Cross) Research Ethics Committee (Reference: 20/LO/0034) approved the APPLE-Tree study in March 2020, and all participants resigned consent forms after an amendment dated December 16, 2021, to include this study. Participants signed consent forms covering potential uses of donated photographs; with further image-specific consent forms for photographs including identifiable images, by all people potentially identifiable. All participants chose to be named in the exhibition beside exhibition pieces, and on a catalog insert. To protect identities, we have altered some minor details of case studies while maintaining the integrity of narratives (Saunders et al., 2015).

### Data analysis

Textual data (transcribed interviews, ethnographic fieldnotes, and display exhibition texts) were analyzed alongside photographs and other visual exhibition works. We drew on Gleeson’s method of polytextual thematic analysis with photographs (2020) and Braun and Clarke’s reflexive thematic analysis (2019), in finding patterns across and between data.

We asked interview participants to take photos that relate to memory concerns and the APPLE-Tree intervention, extending this during the collaborative process to include “things that are important to me.” Although some images shared with us directly engaged with the first two themes, participants primarily shared images, and collaborated to produce artwork, that reflected what was important to them, their everyday lives, and identities. Analysis was guided by looking at how participants used images to express and reflect on their lives, within and across sets of photos, in their accounts and in developing pieces for the exhibition. E. Whitfield and S. Parnell Johnson discussed ideas for themes that appeared to be emerging during the collaborative process, and with other members of the study team. We present overarching conceptual themes, and developed case studies that illustrate how these themes were woven through the participants’ engagement.

## Results

### Sample Description

Table 1 describes characteristics of the 19 participants and how their contributions supported theme development.

**Table 1.** Table of Participant Involvement in the Study

Participant	Age, gender ethnicity, living situation		Study involvement						
		Photo-elicitation interview	Preworkshop individual session	Workshop	Postworkshop individual session	Exhibition coproduction meeting	Exhibition display	Postexhibition interview	Themes (see Notes for key below^b^)
1. Jill	75–84, female, White British, lives alone.	1	2	1	2	2	1	1	1, 2, 3
2. Natasha	75–84, female, White European, lives with husband.	1	2	1	2	1	2	1	1, 3
3. John^a^	65–74, male, White British, lives with wife and adult son.	1	1	1	0	0	1	1	2
4. Christina	75–84, female, White European, lives alone.	1	2	1	2	1	2	0	1, 3
5. Hugh	65–74, male, White British, lives with wife.	1	1	2	0	2	1	1	2, 3
6. Helena	75–84, female, White European, lives alone.	1	2	2	0	0	1	0	1
7. Jessica	75–84, female, White British, lives alone.	1	2	2	2	0	1	1	1, 2, 3
8. James	75–84, male, White British, lives with wife.	1	1	1	1	0	1	1	1, 3
9. Beatrice	85+, female, White British, lives alone.	0	2	1	2	0	2	1	1, 3
10. Sarah	75–84, female, White British.	0	1	2	1	0	1	0	1, 2, 3
11. Robert	75–84, male, White British, lives alone.	0	1	1	1	1	1	0	1, 2
12. Matthew	65–74, male, White British, lives with wife.	0	2	1	0	1	2	1	1
13. Gareth	65–74, male, White British, lives with wife	0	2	1	0	1	2	0	1, 2
14. Christopher	75–84, male, White British, lives with wife.	1	0	0	0	0	Some of this participant’s interview photographs were exhibited as part of a 14th piece designed by the artist. It features photographs from four interview participants who did not take part in the workshops.	0	1, 2
15. Patricia	75–84, female, White British, lives with husband.	1	0	0	0	0	As above.	0	1, 2
16. Lynne	60–64, female, White British, lives with partner.	1	0	0	0	0	As above.	0	1, 2
17. Gaynor	65–74, female, White British, lives with partner.	1	0	0	0	0	As above.	0	1, 2
18. Jane	75–84, female, White British, lives alone.	1	0	0	0	0	0	0	Jane shared three photographs of doors in her house that caused her confusion, in relation to concerns about memory.
19. Afzaa	Unknown, female, Black African, lives with husband.	1	0	0	0	0	0	0	2
TOTAL		14						8	

*Notes:* ^a^John’s work was displayed in an arrangement made by the artist as he could not complete the project due to ill health (see case study).

^b^Key to themes: 1 = Nature as a lifeline; 2 = Family; 3 = Self and identity.

### Analytic Themes

We identified three themes. “Nature as a lifeline” explores how tangible connections to the material anchor and reassure. As illustrated in the case studies, familiar places brought comfort and trees were recurrent emblems of continuity and stability. In a second theme, “Family,” participants portrayed how mutual love and support, past and present, are important to their sense of self and place. Photographs reflected family relationships, including people lost.

“Self and Identity” is defined by reflections on self and identity, enduring through aging, memory concerns, pandemic, and aging stereotypes. This was illustrated in self-reflexive artworks, using memories, experiences, life, and family history to ground and make sense of identity over time.

### Case Studies

Authors discussed and agreed on a selection of four case studies to showcase themes, how our methods and breadth of visual and textual material contributed to their development, interconnections between them, and to illustrate diversity of experiences.

We will draw on the humanities to enhance our understanding of experiences of aging and the visual, making connections to Sara Ahmed’s writing on the power of objects, physical and otherwise, to orient the self; an aspect of participants’ lives that photography makes visible (2006).

#### Jill (illustrating themes 1, 2, and 3)

This case illustrates the power of photographs to pull us into the materiality of a participant’s world. Jill, who is in her late 70s, lives alone. She shared more than 30 photographs in a photo-elicitation interview, which spoke to the intertwining of biography and identity (Frank, 2010), and brought more to her discussions with S. Parnell Johnson.

Her exhibition piece was autobiographical; photographs from her childhood to the present, including images of close family members, mixing sad with happier times. It charted how relationships (Theme 2) and life experiences shaped her personal identity, and how this and her enjoyment of life endured despite memory concerns (Theme 3).

People, places, objects, and photos of art are combined in Jill’s artwork, including photos of herself across the life course (including photos of old photos). This expresses a sense of her “sequential self states,” and an intertwined continuity and discontinuity that is important for photography of aging to engage with (Cristofovici, 2009). The entire artwork, made of many photographs, is an “image of aging” encompassing multiple selves across time.

**Figure 1.**
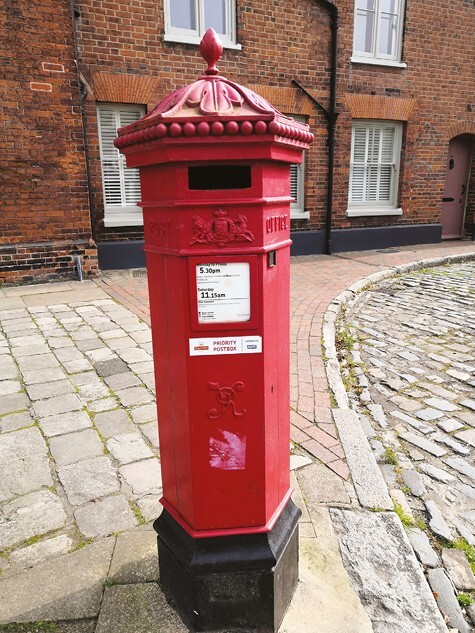
Jill’s postbox photograph.

**Figure 2.**
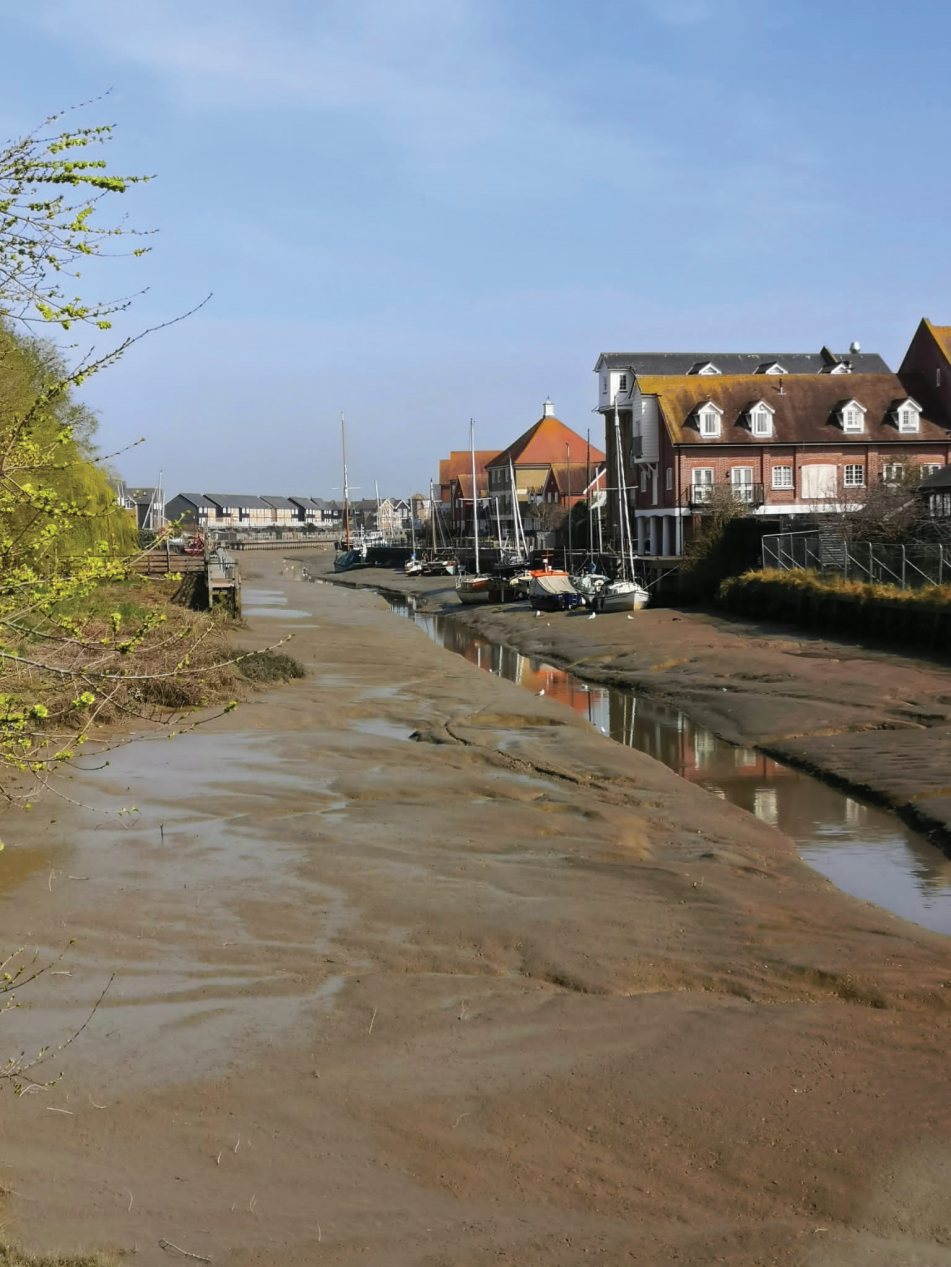
Jill’s creek photograph.

Jill’s artwork celebrated nature and valued the solidity of the material world (Theme 1), connecting it with her pagan beliefs; represented by a photograph of flowers in her garden that are her “saviours.” Her photos included places in her hometown, which evoked important memories and emotion. A postbox reminded her of a relative joking they would post her into it as a child (Figure 1); a local brewery sign evoked memories of familiar childhood smells, and a photograph of a creek of the unfulfilled wish to leave footprints: permanence of nature relative to impermanence of man (Figure 2). She intends her ashes to be scattered there, to “finish where I started.”

These images and reflections speak to an “embodied” sense of place and how memories attach to everyday activities (Pink et al., 2011, pp. 15–16); and to Sara Ahmed’s work on how familiar places and objects can reorientate at times of disorientation, like “a second skin that unfolds in the folds of your body” (2006, p. 9).

Jill’s images reflect resilience (Theme 3). She reflected on physical aging in an image of her hand. Just as Rosy Martin (2002) writes of the “flash of recognition” at seeing her mother or father when she looks in the mirror, Jill felt that it was her mother’s hand she was seeing in front of her, as though past generations are embodied in aging selves.

She likened herself to the poppies in one of her photos, which stood tall when the plants around them had wilted. She stated in a text for the exhibition that the possibility of future memory loss is her next “challenge”:

My life has consisted of fantastic highs but many, many lows from which I have fought back from with a vengeance in the past, this may be more of a challenge, but I am sure as hell going to try as there is so much more I want to do.

A photograph of her shadow taken during the pandemic represents fears of becoming “a shadow of my former self.” She recalled how her voice had changed with less use, and isolation had affected her memory. She is “throwing” herself into activities now and described her exhibition work as an exhortation for people to fight against what might be “round the corner,” reflecting the “event horizon” of the fourth age (Gilleard & Higgs, 2010).

Her exhibition piece reflected a playful, “theatrical” personality. Photos were printed onto a magnetized board, with her sticky notes as vinyl pieces so that the audience could move them and some images resisted or played with age-related stereotypes, such as a photograph of her dancing. She embraced this idea, which reflected a resilient identity based on deep connections to the material world, within shifting scenes in life. Opposing stereotypes can be inadvertently reproduced in research seeking alternative images of aging (Parnell Johnson, 2018; Richards et al., 2012). Jill’s exhibition piece provides a more complex, layered, and balanced image of aging. The photograph of her shadow and references to concerns about memory conveyed authentic vulnerability, contextualized in images of humor and active embracement of life.

Her artwork, entitled “Uncertainty,” was brave and moving and attracted a lot of interest; Jill “really enjoyed” explaining it to people. She reflected after the exhibition on how her “higgledy-piggledy” arrangement reflected the course of her life, looking back. She felt that the project was cathartic and it seemed to enable what Habermas and Köber (2015) termed “autobiographical reasoning,” described as “a process of thinking or speaking that links distant elements of one’s life to each other and to the self in an attempt to relate the present self to personal past and future” (p. 666), and which is suggested to support a “sense of self-continuity” across time.

#### Beatrice (Themes 1 and 3)

Beatrice’s work celebrated the natural world (Theme 1), and the development of new rituals that enhance older age (Briller & Sankar, 2011). She used images to express resistance to aging stereotypes and saw in the project the capacity to revitalize and offer new hope, a source of resilience in itself (Theme 3). Beatrice is in her early 90s and a retired secretary, she completed an art degree in her 70s and continues to paint and display work. She was interested to learn techniques from S. Parnell Johnson and donated more than 200 photographs.

**Figure 3.**
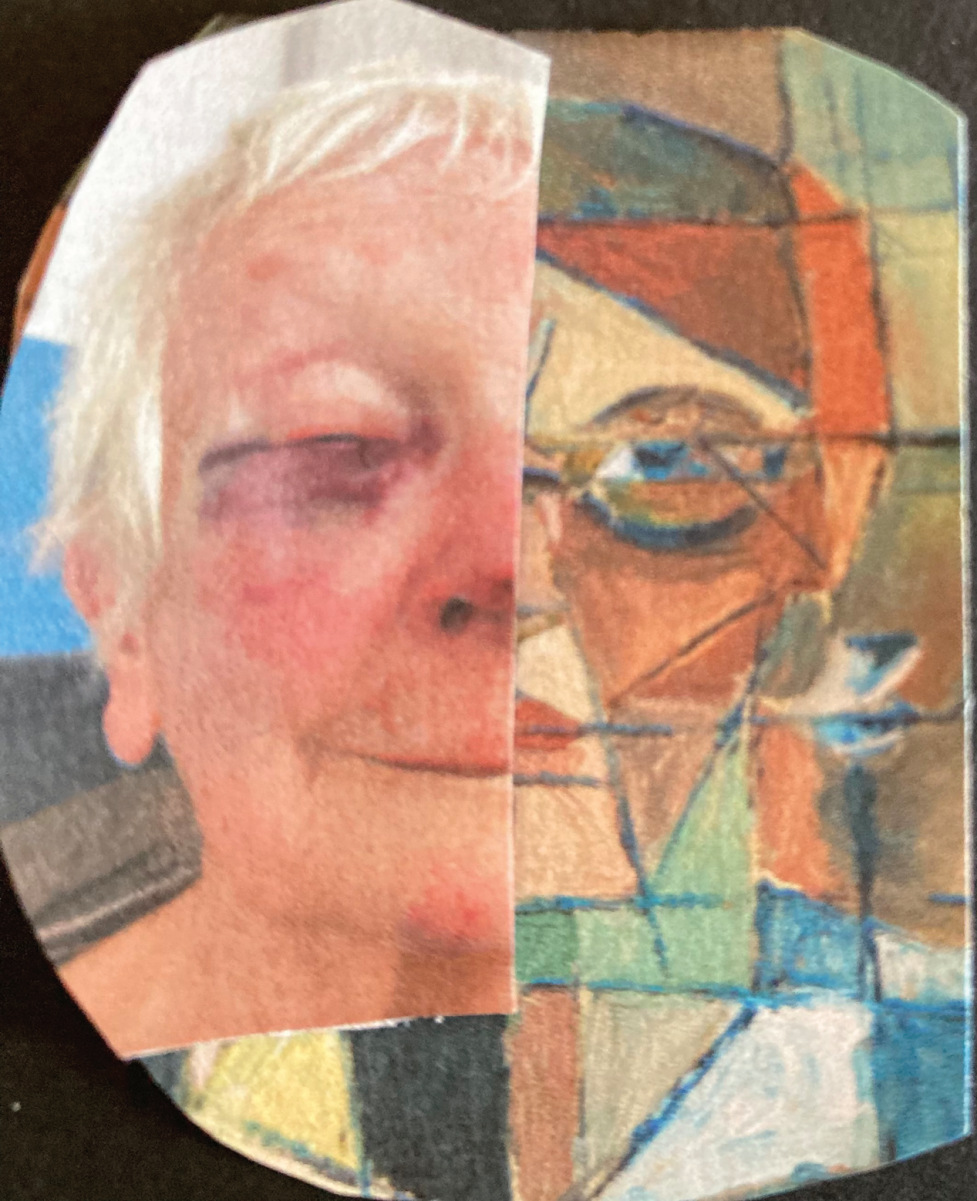
Beatrice’s photomontage self-portrait.

The first images that Beatrice shared included two of herself: one in sunglasses and another with a badly bruised face from falling a few weeks after her 90th birthday. In her plans to use this powerful image of vulnerability for a painted self-portrait, she illustrated creativity in adversity, confronting her experience of vulnerability with resilience through art. She later sent us this image within a cubist-style self-portrait (Figure 3). While Jill presented separate images in conjunction on a backdrop, here Beatrice brings together two selves in a compound split image of aging. It is a composite diptych photomontage image, with two faces split centrally and joined as one. One half represents her identity as a painter, the other a vulnerability (at least partly) subverted. It seems to indicate agency in the face of adversity, interrupting readings of creative decline and stasis, in images of older women particularly. She reflected that “it sometimes seems that one is flaunting to overcome the reluctance to show oneself? It could be that as one gets older defiance sets in and you need to prove (also to yourself) that you are still around and alive!”; her own “challenge” to the invisibility of older women (Martin, 2002).

**Figure 4.**
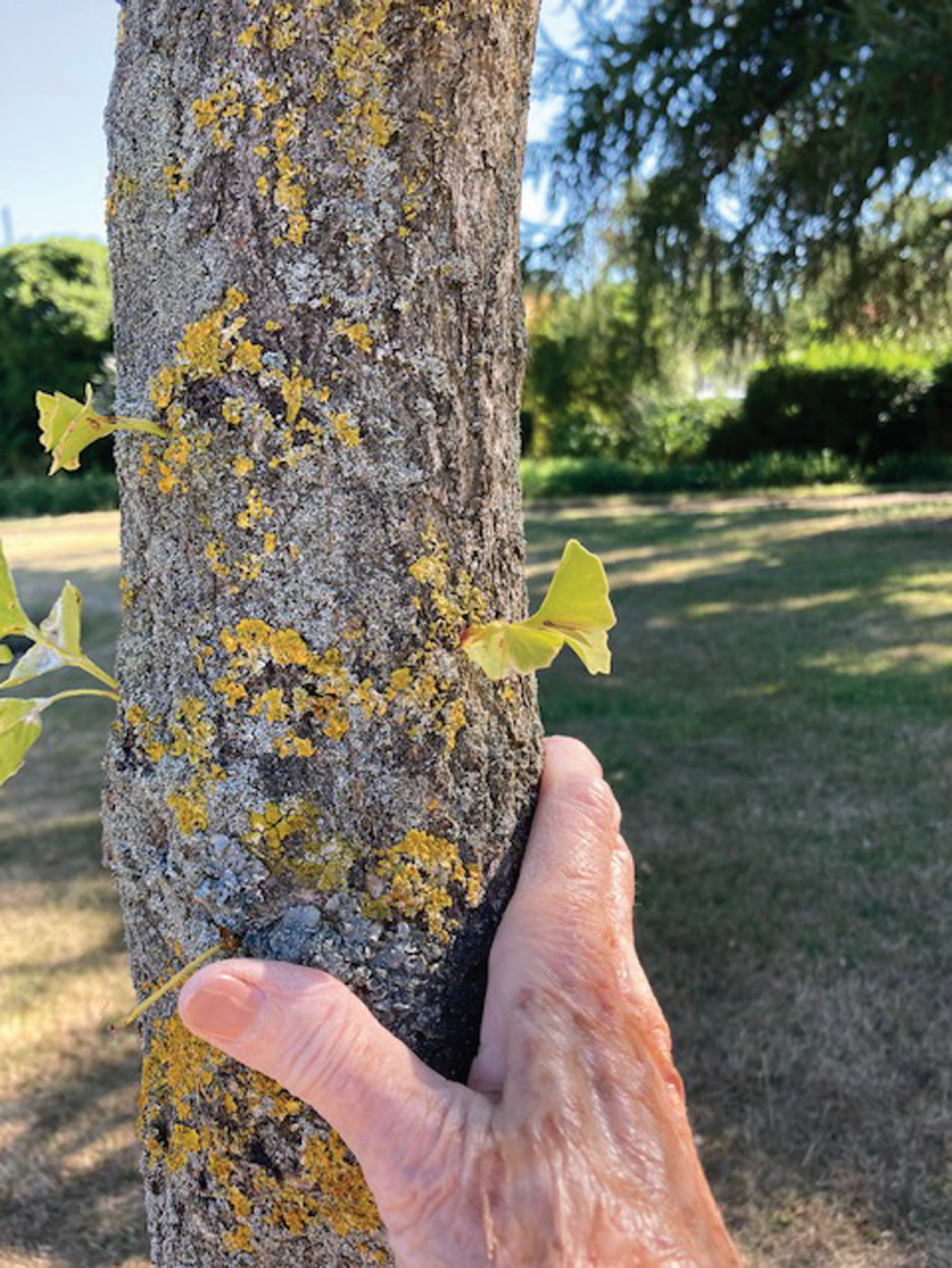
Beatrice’s ginkgo tree.

Beatrice lives alone and during the pandemic lockdown, she was afraid to go out. Walking in the local park early one day when it was quiet, she photographed a ginkgo biloba tree. She has photographed it many times since, from different angles and in different seasons (Figure 4), and experimented with photographing its leaves. She refers to it as “my ginkgo” and also connects it to when she began taking ginkgo biloba tablets due to concerns around memory. It has represented hope and ontological security (Giddens, 1991) in a time of disorientation. She found a sense of continuity and resilience in it, saying “It seemed to inspire me you know, sort of keep going and seeing it, you know, as long as you see the tree you’re going to be all right,” and:

its roots there and, you know, as I said, if it’s bare or green, you know, when it loses its leaves, it’s still there and I feel happy about it, it varies. It’s OK, things are going to be OK, fine. Life is good, it keeps me going I think that tree

Beatrice sent us many photographs of the tree over the course of the project and we emailed feedback and encouragement. Visiting it was a ritual or “refrain” through which a person’s sense of “going on being” is “held” (Walkerdine, 2013, drawing on Guattari) and speaks to the enhancing power of nature (Orr et al., 2016). She described it as a “lifeline,” a word we used in our title and that Ahmed uses to describe an object, or sense of possibility, that can decrease disorientation; something that can be grabbed hold of, physically or figuratively, to help someone back on “course” or into feelings of safety (2006). Beatrice described an embodied affinity with the physical form of the tree, “They’re rather like an old body, an ageing body and you think it looks OK, you know, you love it but really it’s changed.” S. Parnell Johnson created a book of her photographs for the exhibition, including multiple images of the tree, the bruised face photos, and the split portrait.

**Figure 5.**
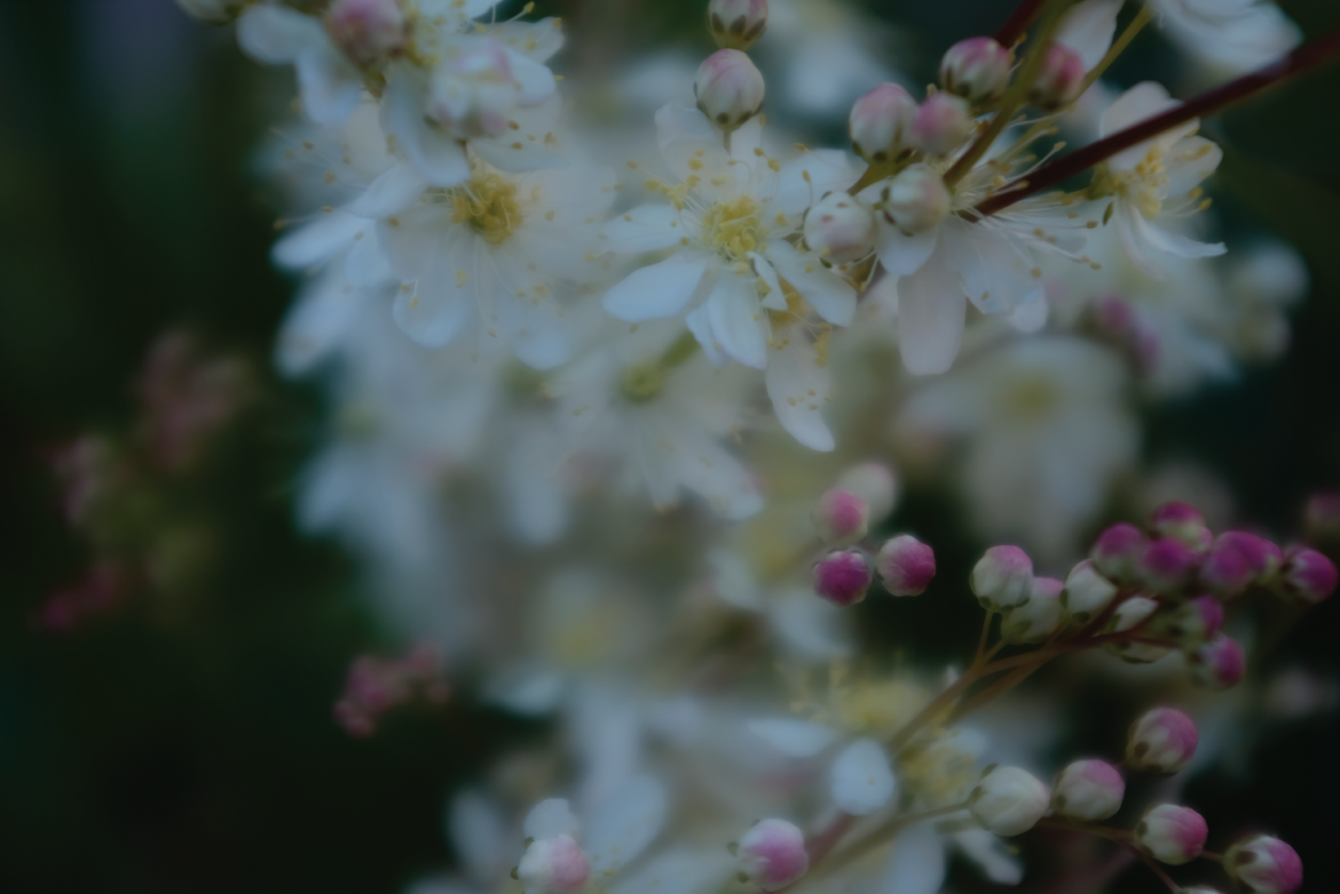
Beatrice’s apple blossom.

Beatrice displayed a photograph of the apple tree in her garden in the exhibition, taken with a new skill of using a short depth of field that blurs the background, to express her thoughts about the mind and memory (Figure 5). She described it as showing “a mind which is blurred by the memories of one’s life” but with the buds opening “like synapses,” leading to “the joy of the rosy apple to be tasted later in life.” Her text for the exhibition was titled “Photographs Ignite Memories.” Here is an excerpt:

Early one Sunday walk, I noticed a Ginkgo Biloba tree in my local park. Taking a photo of it to send to my daughter proved I had been out for the necessary walk. This unique “living fossil” has become an important lifeline for me. Every time I photograph “my ginkgo” I see something different about it! The camera’s “blurring eye” made my brain think again—how to? With so many photos taken, I found it difficult to eliminate as each one was a moment taken in my life. Every photo was meaningful. My synapses fired—I made some collages. How to arrange some of the leaves and photograph them? Haiku writing kicked in, another way of expressing joy!

A family history of Alzheimer’s, and memory concerns, motivated Beatrice to take part in APPLE-Tree. It was clear how much she valued the photography project. She described actively exploring connections between current and past images through her visual imaginary, writing “My brain is ignited and I remember the energetic days of Uni again”; and “I no longer feel afraid.” This speaks directly to Ahmed’s work describing how people turn away from fearful objects and towards positive possibilities. Bringing Beatrice’s words together with Ahmed’s theory highlights the disorientating effect of fear, with the project as a reorientating object of hope that created a new sense of possibility (2006), via the visual imaginary, reflections, and supportive and collaborative relationships.

The image of her hand touching the ginkgo tree (Figure 4) shows continued engagement with the material world beyond the confines of home, and an embodied affinity with nature and this tree in particular, in which she sees a reflection of her own (aging) body. In the technically accomplished image of apple blossom (Figure 5), and words that accompanied it, we see the expression of an identity as a skilled photographer, actively engaged with the world and with a continued capacity for self-development that can be seen in the photograph’s visual and aesthetic effect.

Beatrice was proud to exhibit her work and ordered multiple copies of her print and book for family/friends. Her work shows the transformative potential for this kind of collaborative project, as she embraced the ongoing creation of her own subjectivity (Pink et al., 2011), which the project allowed us to both evoke and capture.

#### Natasha (Themes 1 and 3)

Natasha is in her early 80s and lives with her husband. Originally from Hungary, she enjoys art galleries and belongs to a photography club. Natasha has word-finding difficulties and mobility problems following a stroke. Ahmed (2006) describes how objects, thoughts, and ideas, can succeed or fail at “extending” us into the world. For Natasha, words could not always be relied upon for this type of extension.

Natasha shared photographs from her large existing collection. She approached these thematically and used symbolism, often giving them titles and abstract meanings. In early sessions, she and S. Parnell Johnson discussed images that spoke to bodily and health constraints, aging, memories, feelings of being blocked and curtailed, and dichotomies of choice. A tree in winter represented death. She also expressed a love of color, structure, and light as positive elements in images.

**Figure 6.**
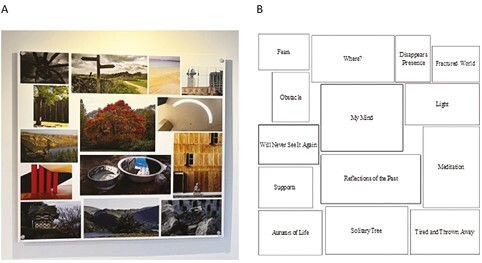
Natasha’s “Reflections and Imaginations”. (A) An image of the piece in the exhibition. (B) An image of the corresponding table.

Her final piece, “Reflections and Imaginations” (Figure 6), includes visual metaphors of the aging process. The nebulous tree in the center, entitled “My Mind,” reflects current challenges; a pair of old shoes called “Tired and Thrown away” symbolize her aging identity; and a fence symbolizes obstacles, visually barring the way for both photographer and viewer. A photo of a place she has visited in the past is called “Will Never See It Again,” and a view from below a huge spider sculpture against a darkening sky, suggesting overwhelm, is called “Fears,” (all in Figure 6). She also photographed a Louise Bourgeois sculpture called “The Mute,” reflecting feeling “lost for words” and shared images of Bourgeois’ “Spider” sculpture, commenting on the fragile legs, symbolizing her fear of falling and breaking bones. These speak directly to the aging body without depicting it.

Her symbolic use of scenes and objects to represent aging and physical constraints renders her physical form invisible and externalizes her affective experiences. These images speak to Natasha’s negative experiences of aging whereas the artistry brings a form of comfort. It also shows how photographers can depict internal responses to aging, finding novel ways to express “the inner screen of aging” (Cristofovici, 2009, p. 18).

Her accompanying text (“thoughts about getting older”) described the constraints of aging and thought of death. She described how colors and symbols have become a great consolation to her in later years:

For several years I have been interested in photography, films and arts in general. In my years now they are like a raft that keeps me afloat and happy to deal with shapes, forms, colours. What they mean, and their symbolism are with me and make my life bearable.

Art mixes with science or even theology and all that begins to mean more to me now, than in my youth. Ideas and messages are appearing in images, there are so many meanings to everything if given a chance.

The arts seem to act as her anchor in a world that feels more restricted.

Natasha used a powerful image in her postexhibition interview to communicate her experience of pain: a postcard of an Antony Gormley sculpture of a suspended figure stabbed with nails, which showed how she feels when walking, “because everything aches when I am walking.” This is reminiscent of Deborah Padfield’s work on the therapeutic value of expressing inarticulate pain via photographic images (2021).

Her photographs and reflections speak to the importance of understanding how older people experience suffering and pain and what modulates or mediates this experience; representing or communicating the experience of suffering through language is an area that presents a particular challenge within gerontology (Medeiros & Black, 2015).

#### John (Theme 2)

John is in his late 60s and lives with his wife Helen and adult daughter. John spoke of the reciprocity and depth of love in his relationship with Helen, who accompanied him to the workshop. John took many images of Helen during the project but deleted them as none represented his internal image, a palimpsest softened and shaped by years of memories (Parnell Johnson, 2018). John decided, after discussion with S. Parnell Johnson, to withhold Helen’s portrait from view by turning it over and presenting the back of the image captioned with his loving feelings towards her. In suggesting this, S. Parnell Johnson drew on visual strategies that she had developed in earlier work around withholding images of older women, based around Barthes’ withholding of his mother’s image in Camera Lucida (2018).

Unfortunately, John left the project early due to illness but said that he had “really enjoyed” the memories that it brought up. With his support, S. Parnell Johnson created a piece based around photos of his children and grandchildren and the reversed photo of his wife, using his images and plans. He wrote text to display alongside, beginning:

My family has always been the most important thing to me.

My children and grandchildren are so very important to me; I remember where and when I first met my wife, I remember all my children being born and the first loving words I said to them all.

It described familial love, including love from parents and between siblings when he was a child, as a point of continuity throughout life, and spoke to creative ways of evoking multiple “sequential self states” over time (Cristofovici, 2009). Photographs of John’s notes on love and family formed a backdrop to the photos within the artwork, and accompanying text acted as an “elaboration” of the work (Kress & Leuwen, 2006, drawing on Barthes).

Generations, in particular grandparenthood, was an important theme in another photography exhibition looking at “New Images of Age(ing)” (Staudinger, 2011), often through images of grandparents alone or with grandchildren or great-grandchildren. Here, the relationships between generations, and John’s identities as husband, father, grandfather, child, and sibling, were evoked by bringing together multiple photographs and pieces of text into one artwork with a further accompanying text. It referenced multiple familial relationships past and present, with John himself implicit, an absent presence behind the camera, in a work that the audience found very moving.

### The Exhibition

Exhibition artwork spoke to questions of nature and place, the importance of relationships, biography, identity, generation, and the aging process itself. Participants captured their own lived experiences and reflections, where aging was not necessarily foregrounded but was implicit. It was important to honor the artworks by staging the exhibition imaginatively and professionally. Artworks were accompanied by text that deepened the audience’s understanding of their meaning and by participants’ reflections on the APPLE-Tree intervention. Attendees spoke of the insight the rich and diverse pieces gave them into participants’ lives.

**Figure 7.**
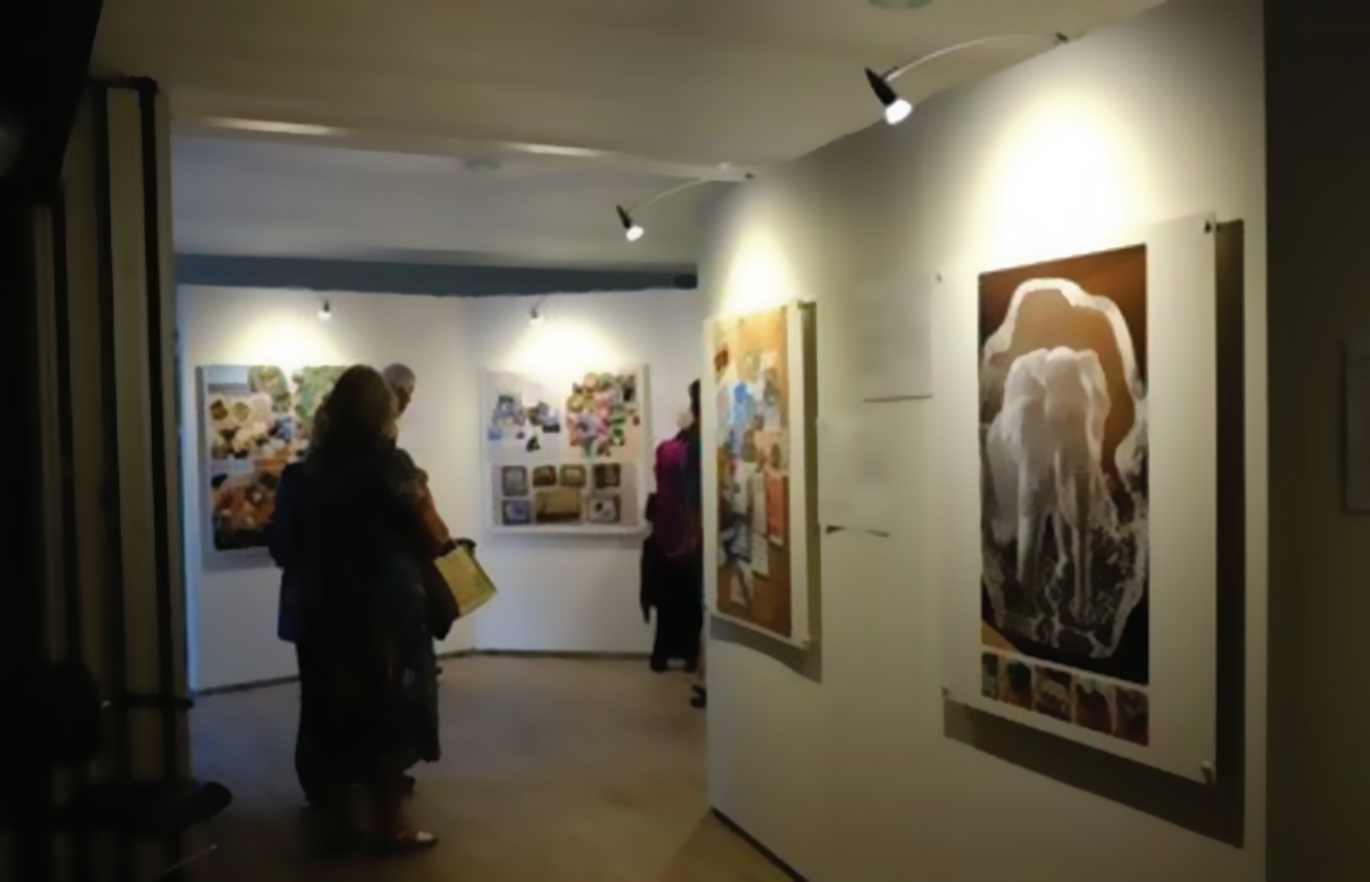
Attendees at the exhibition in the Wellcome Collection.

The exhibition, titled “Reimagination: The reframing of Memory,” was initially displayed for one day at the Wellcome Collection in London (September 8, 2022), attended by around 200 people (Figure 7). Photographs from 17 participants were displayed, including a piece designed by S. Parnell Johnson using images from four interview participants who had not taken part in the workshops (Table 1). She also worked with participants to develop original display formats. A piece relating to time was arranged in a circle with a central three-dimensional clock mechanism pointing to individual images (James). A piece celebrating family, community, nature, and place was displayed on a map, with pins and string plotting the participant’s habitual routes (Robert). Two participants displayed photo books near their artwork, exploring themes that included nature and embodiment (Beatrice and Christina). S. Parnell Johnson created a praxinoscope (circular spinning device) for a participant who had taken many photographs of the same building at different times of day (Matthew). One participant lent the exhibition a memory book that his family had made for his late father, which was displayed on a podium. Ten exhibitors attended the launch with family and friends. We produced one short film while creating the exhibition and one of S. Parnell Johnson giving a tour of it. The exhibition was also displayed in the Houses of Parliament for a week, then at Holy Sepulchre London church (February–April 2023), after which we offered the artwork to participants to keep.

The therapeutic potential in relation to memory concerns, of working with a professional artist/photographer in this way, is reflected by Beatrice, who told us how the project helped her to move past her fear of memory loss. Another participant described how it helped her resolve to persevere with challenges, suggesting an increased confidence. She has used it to discuss her memory issues with friends, showing them images from the exhibition.

## Discussion

We have presented themes developed through participant engagement with the project, in which photographs were seen to celebrate connections to nature as a lifeline; anchor lives within the context of relationships with family; and reflect on self and identity, enduring through aging, memory concerns, pandemic, and aging stereotypes.

We described how creative collaborative photography can help in accessing interior worlds, thoughts and feelings, and be an important, positive experience for participants, with potential to create the space to “embrace creative, reflexive, complicated selves,” as described by Winton (2016, p. 428).

Working with the artist/photographer individually and in small groups produced images and artworks that gave new insights into ways of representing and exploring aging, from the subjective perspective of older people. This contrasts with projects where representation is directed by a professional photographer and the rich diversity of participants’ experiences can be overlooked (Richards et al., 2012). In liaison with the artist/photographer, participants transformed data from social science into art that enabled audiences to question and challenge their own ideas and stereotypes around aging, memory, and everyday life. This suggests the importance of participatory photography, across gerontology, not only in creating discursive data but also in the power of the images themselves. Using visual methods in this sustained collaboration allowed insights into participants’ worlds that may be invisible to other methods.

The photographs and other data discussed here speak to important areas within social gerontology as well as age studies, including ageism (Goldman & Higgs, 2021), the embracing of third age identities and fear of the fourth age (Gilleard & Higgs, 2010; Gilleard et al., 2005), the body, embodiment, and embodied practices (Gilleard & Higgs, 2013; Martin & Twigg, 2018; Twigg & Martin, 2015a, 2015b), resistance to stigma and invisibility (Hogan & Warren, 2012; Martin, 2002, 2015), and the importance of place and aging in place (Pani-Harreman et al., 2021). We have illustrated the breadth of data that can be generated by participatory photography and, in particular, the kind of imagery that can be produced, speaking to the heart of cultural gerontology and to the aim of “refreshing the gerontological imaginary” (Twigg & Martin, 2015a, p. 357).

Completing this project and reflecting on the artwork and reflections produced from it, we wonder what more could be done with this method, and how it might be extended to allow more time and opportunities to explore the creative potential of using photographs in conversation with an artist/photographer and other participants; further reflections on aging identities may emerge from this process.

### Limitations

There was inevitably an element of self-selection in those who took part. We separated recruitment for the interviews and the exhibition to try to broaden participation.

Unfamiliarity with technology can be a potential obstacle in using these methods with older people (Mysyuk & Huisman, 2020); use of email, Zoom, a camera or other device, and ability to send images over email were essential. Some participants asked friends and family to assist with sending photographs, and we helped to download or email images during workshops, if required.

The majority of participants were White British and most of those from minority ethnic groups were White Europeans from outside the UK. This reflected the make-up of the sample from which we recruited (the APPLE-Tree intervention). There were two further interviews with participants from other minority ethnic groups, but as they did not share photographs we did not include them in this analysis. It is likely that participants had greater cultural capital than those who declined to participate and this helped to shape their engagement. Most did not have an arts background, though Beatrice and Natasha did have some previous involvement in arts creation.

## Conclusion

This article has reflected on the use of participatory photography with participants with memory concerns engaged in a dementia-prevention study. It has traced the process through photo-elicitation interviews, collaborations in zoom sessions, workshops, coproduction meetings, and a cocreated exhibition. It has shown the creative possibilities that this can engender and capture, the potential to generate rich data that enhance the study of aging, and the positive effects that can be experienced by participants. Moreover, it demonstrates the rich cross-fertilization that engagements between the arts and humanities and gerontological research can have in extending our understanding of the lived experience of later life in its myriad forms.

## Data Availability

Data are not available to other researchers for replication purposes due to ethical reasons relating to the potential identification of participants. We are also not making analytic materials available as we intend to continue with analysis for the purpose of further publications. This research was not preregistered with an independent institutional agency. London (Camden and Kings Cross) Research Ethics Committee (Reference: 20/LO/0034) and UK Health Research Authority (HRA) approved the APPLE-Tree study in March 2020. This was amended on December 16, 2021, to include this study.
